# Mechanisms of Graft-versus-Host Disease Prevention by Post-transplantation Cyclophosphamide: An Evolving Understanding

**DOI:** 10.3389/fimmu.2019.02668

**Published:** 2019-11-29

**Authors:** Natalia S. Nunes, Christopher G. Kanakry

**Affiliations:** Experimental Transplantation and Immunology Branch, Center for Cancer Research, National Cancer Institute, National Institutes of Health, Bethesda, MD, United States

**Keywords:** post-transplantation cyclophosphamide, haploidentical allogeneic hematopoietic cell transplantation, graft-versus-host disease, skin allograft rejection, alloreactive T cells, tolerance, regulatory T cells

## Abstract

Post-transplantation cyclophosphamide (PTCy) has been highly successful at preventing severe acute and chronic graft-versus-host disease (GVHD) after allogeneic hematopoietic cell transplantation (HCT). The clinical application of this approach was based on extensive studies in major histocompatibility complex (MHC)-matched murine skin allografting models, in which cyclophosphamide was believed to act via three main mechanisms: (1) selective elimination of alloreactive T cells; (2) intrathymic clonal deletion of alloreactive T-cell precursors; and (3) induction of suppressor T cells. In these models, cyclophosphamide was only effective in very specific contexts, requiring particular cell dose, cell source, PTCy dose, and recipient age. Achievement of transient mixed chimerism also was required. Furthermore, these studies showed differences in the impact of cyclophosphamide on transplanted cells (tumor) versus tissue (skin grafts), including the ability of cyclophosphamide to prevent rejection of the former but not the latter after MHC-mismatched transplants. Yet, clinically PTCy has demonstrated efficacy in MHC-matched or partially-MHC-mismatched HCT across a wide array of patients and HCT platforms. Importantly, clinically significant acute GVHD occurs frequently after PTCy, inconsistent with alloreactive T-cell elimination, whereas PTCy is most active against severe acute GVHD and chronic GVHD. These differences between murine skin allografting and clinical HCT suggest that the above-mentioned mechanisms may not be responsible for GVHD prevention by PTCy. Indeed, recent work by our group in murine HCT has shown that PTCy does not eliminate alloreactive T cells nor is the thymus necessary for PTCy's efficacy. Instead, other mechanisms appear to be playing important roles, including: (1) reduction of alloreactive CD4^+^ effector T-cell proliferation; (2) induced functional impairment of surviving alloreactive CD4^+^ and CD8^+^ effector T cells; and (3) preferential recovery of CD4^+^ regulatory T cells. Herein, we review the history of cyclophosphamide's use in preventing murine skin allograft rejection and our evolving new understanding of the mechanisms underlying its efficacy in preventing GVHD after HCT. Efforts are ongoing to more fully refine and elaborate this proposed new working model. The completion of this effort will provide critical insight relevant for the rational design of novel approaches to improve outcomes for PTCy-treated patients and for the induction of tolerance in other clinical contexts.

## Introduction

Hematopoietic cell transplantation (HCT) is the only potentially curative treatment for many patients with advanced hematologic malignancies or severe non-malignant diseases. Human leukocyte antigen (HLA)-matched donors are the historical gold standard source for HCT, but they are unavailable for many patients, including the majority of those not of white European ethnicity (1). The use of donors who are not fully HLA-matched has been associated with high levels of graft-versus-host disease (GVHD) and graft rejection (2, 3), which are attributable to strong bi-directional responses of donor and host alloreactive T cells (3, 4). Fortunately, newer strategies have improved outcomes for HCT using partially HLA-mismatched donors, resulting in outcomes comparable with those seen with HLA-matched donor HCT (5).

Among these strategies, post-transplantation cyclophosphamide (PTCy) has become widely used as GVHD prophylaxis (6). A research group from Johns Hopkins University was the first to utilize high-dose PTCy in their platform for HLA-haploidentical bone marrow HCT (7, 8); PTCy since has been extended to other HCT platforms and a variety of donor types (6, 9). Accelerating its widespread adoption is the fact that PTCy is an inexpensive treatment that does not require extensive training for its administration, thus resulting in high accessibility. Furthermore, clinical outcomes using PTCy have been quite promising; registry data suggest that PTCy reduces chronic GVHD incidence after HLA-haploidentical HCT, resulting in decreased GVHD rates but similar survival compared with patients undergoing standard HCT using HLA-matched related or unrelated donors (10–13). The benefits of PTCy in preventing severe acute and chronic GVHD observed in the HLA-haploidentical HCT setting also have been found in the HLA-matched donor HCT setting (14–16), further decreasing the need for other post-transplant immunosuppression (17).

Recently, in a prospective multi-center, randomized phase II clinical trial, Bolaños-Meade and colleagues have compared the relative efficacy of three novel approaches for GVHD prophylaxis after reduced-intensity conditioning, HLA-matched related or unrelated donor HCT (18). Each of the three novel agents was added to standard calcineurin-inhibitor (CNI)-based GVHD prophylaxis, and each regimen was compared to a contemporaneous non-randomized control group receiving standard tacrolimus and methotrexate (18). Only the PTCy-containing regimen resulted in superior GVHD-free, relapse-free survival (the primary endpoint). The PTCy-containing regimen also provided superior protection against both severe acute GVHD and chronic GVHD requiring immunosuppressive therapy (18).

While we still await the results of ongoing and planned randomized phase III studies, PTCy already has had a dramatic impact on the HCT field. Rates of HLA-haploidentical HCT have risen precipitously, and the use of PTCy steadily has been making in-roads into HLA-matched HCT, which will likely accelerate given the results from the randomized phase II study detailed above. Yet, in order to rationally improve upon outcomes seen with HCT using PTCy, it is of the utmost importance to have a detailed understanding of the mechanisms by which PTCy prevents GVHD after HCT. It has been long believed that we understood these mechanisms based on extrapolation from major histocompatibility complex (MHC)-matched murine skin allografting models using cyclophosphamide (19, 20). However, these mechanisms have not fit with some of the clinical observations in human HCT (e.g., no substantial impact of PTCy on grade II acute GVHD), and recent data suggest that these mechanistic explanations may not be true in murine HCT models (21–23). Therefore, this review provides an overview of the history of cyclophosphamide's use in preventing skin allografting rejection experimentally and of the evolving understanding regarding mechanisms of GVHD prevention by PTCy after HCT.

## Early Studies of Cyclophosphamide as a Tolerogenic Agent

There is an intriguing duality in the history of cyclophosphamide as it has been used both as a pro-inflammatory and as a tolerogenic agent (24). The latter has taken many forms experimentally, including prolongation of skin allograft survival, mitigation of GVHD, and prevention of antibody-mediated responses to antigens, such as erythrocytes (25–35).

In the early 1960s, Berenbaum and Brown began to investigate the use of cyclophosphamide to prolong skin allograft survival in an MHC-haploidentical murine experimental model. They observed that a single high-dose injection of cyclophosphamide given during the critical window of days 0 to +4 was able to delay skin allograft rejection (25). Owens and Santos found in a partially-MHC-mismatched murine HCT model that when cyclophosphamide was given at 37.5 or 75 mg/kg/day on days +5, +8, +11, and +14, it was successful at preventing fatal GVHD, distinct from 6-mercaptopurine, methotrexate, cortisol, or mechlorethamine, which were all ineffective (33). Although protected from fatal GVHD, cyclophosphamide-treated mice still rejected skin allografts from the host or third-party strains, but with slower kinetics than untreated mice, suggesting that alloreactivity against host antigens was not lost after cyclophosphamide. Moreover, in a rat HCT model, low-dose (5–10 mg/kg/day) cyclophosphamide was successful at preventing fatal GVHD when given on days +2, +3, and +5, but was ineffective if begun later, starting on day +7 (35). Overall, these several studies showed that PTCy could exert a tolerogenic effect on allograft survival or GVHD when given early post-transplant.

In an attempt to improve murine skin allograft survival, Nirmul and colleagues (1973) introduced a new concept in which they gave priming allogeneic MHC-haploidentical splenocytes on day 0, cyclophosphamide on day +2, and skin allografts (from the same donor strain as the splenocytes) on day +12 (34). This approach produced a 2-week prolongation of skin allograft survival, although these grafts still were rejected (34). These investigators also began to more thoroughly investigate the timing and dosage of cyclophosphamide, observing that a 200 mg/kg single dose on day +2 was superior in effect to the same total dose divided in smaller doses over days +2 to +5. They also found that the dose of splenocytes administered was important, as a dose of 100 x 10^6^ cells was more effective than 50 × 10^6^ cells (34).

## Efficacy of Cyclophosphamide in Preventing Rejection of MHC-matched Skin Allografts

In a series of 13 papers from 1984 to 1987, a Japanese investigative group from Kyushu University continued to study and develop the approach pioneered by Nirmul and colleagues. At first, they evaluated the differences between tumor and skin allograft survival in models like Nirmul's, in which viable donor splenocytes were given prior to cyclophosphamide administration. Consistent with previous data on the optimal timing of cyclophosphamide in skin allografting models (34), MHC-mismatched tumors continued to grow if cyclophosphamide was given between 1 and 3 days after splenocyte administration, whereas accelerated rejection was seen if cyclophosphamide was given on day −2, 0, +5, or +7 in relation to splenocyte administration (36). Tolerance was antigen-specific, however, as injection of tumors of a different MHC-mismatched strain were rejected even with cyclophosphamide. Furthermore, prevention of tumor rejection required giving viable donor splenocytes at a specific dose, as simply infusing large amounts of soluble donor antigen was ineffective (36).

Surprisingly, MHC-mismatched skin allografts would be rejected in models in which MHC-mismatched tumors were not rejected, suggesting critical differences between responses to the two types of allografts (36). Furthermore, the dose of MHC-mismatched tumor given to cyclophosphamide-treated mice was important as small doses were rejected rapidly, while large doses of tumor were not rejected (37). Despite continued tumor growth, mice treated with cyclophosphamide retained antigen-specific cytotoxic lymphocyte responses to tumor alloantigen after cyclophosphamide as measured via delayed footpad reaction and chromium release cytotoxicity assays, and there actually was heightened reactivity early after cyclophosphamide treatment (38).

However, a very different effect was seen after MHC-matched skin allografting. In this setting, survival of skin allografts was substantially prolonged in cyclophosphamide-treated mice, which was accompanied by abrogated responses in delayed footpad reactions and cytotoxicity assays (39). Prolonged skin allograft survival also was seen in syngeneic sex-mismatched transplants, in which male skin allografts were not rejected by female hosts when primed with male splenocytes beforehand and given cyclophosphamide on day 0 (40). Other differences were observed between the MHC-matched and MHC-mismatched systems. Thymectomy had no effect in MHC-mismatched models (41), but resulted in decreased skin allograft survival in MHC-matched models (42). Conversely, splenectomy could prolong skin allograft survival somewhat in the MHC-mismatched models but had no effect in MHC-matched models (42).

This group spent a considerable amount of time better developing and understanding their model system. They found that the activity of cyclophosphamide in MHC-matched skin allografting peaked when given on days +2 or +3 after infusion of the priming splenocytes, as measured by skin allograft survival, delayed footpad reactions, and cytotoxicity assays (43). The cyclophosphamide dose seemed important as it was marginally less effective when given at 150 mg/kg than at 200 mg/kg (43). The dose and type of cells also were important; if thymocytes or bone marrow cells were used as the priming cells, cyclophosphamide was much less effective (43, 44). Yet, the presence of viable donor stem cells either from spleens or bone marrow was necessary for successful maintenance of MHC-matched skin allograft, and some degree of donor engraftment and mixed chimerism in the thymus was critical (44, 45). Irradiating the priming donor cells abrogated the tolerogenic effect, and lower doses of cells actually led to accelerated rejection (44). The age of the recipient, but not the donor, was crucial; there was no significant difference between recipients of 6–12 weeks of age, but reduced tolerance to the allograft was observed when recipient mice were 40 weeks of age (46). Thus, the development of this model identified several crucial parameters needed for successful prolongation of skin allograft survival by cyclophosphamide, including MHC-matched donor/recipient pairs, specific dose and timing of PTCy, specific dose and type of priming donor cells, achievement of intrathymic mixed chimerism, and specific age of the recipient.

Later efforts were focused on trying to overcome the barrier to MHC-mismatched skin allografting. Two successful approaches were identified. The first consisted of T-cell depletion with anti-Thy1.2 antibody treatment on day −1, transplant of spleen and bone marrow cells on day 0, followed by high-dose cyclophosphamide on day +2 (47). Another method successful in overcoming the barrier to MHC-mismatched skin allografting involved two rounds of cyclophosphamide, which could successfully first induce tolerance across major MHC antigens and then secondarily across minor antigens (48).

## Proposed Mechanisms by Which Cyclophosphamide Prevented Rejection of MHC-matched Skin Allografts

This group proposed that three mechanisms mediate prevention of MHC-matched skin allograft rejection by cyclophosphamide (49). The first mechanism, thought to be the dominant one, was described as direct deletion by cyclophosphamide of highly proliferating host mature T cells that were alloreactive to donor antigens. The second mechanism was proposed to be intrathymic clonal deletion of donor-alloreactive host precursor T cells, and the third mechanism was proposed to be induction of host suppressor T cells (49).

Regarding the first mechanism, the authors showed that in the MHC-matched setting, splenocytes derived from tolerized mice at day +35 were unresponsive *in vitro* to stimulation from cells from the priming strain, whereas they responded normally to third-party antigens (50). They hypothesized that this unresponsiveness was due to selective elimination of alloreactive T cells by cyclophosphamide. To test this hypothesis, they leveraged mismatches within the minor lymphocyte stimulating system [responses to proviruses of the mouse mammary tumor virus incorporated into the genomes of certain mouse strains (51)] between different mouse strains to provide markers of alloreactive T cells. In their MHC-matched models, mixed chimerism was established in the lymph nodes by day +14 (49). At that time point in the lymph nodes, there was a substantial two-thirds reduction in the percentages of CD4^+^ T cells, but not CD8^+^ T cells, that were donor-alloreactive (Vβ6^+^); there was continued decline through days +35 and +70 in the percentage of CD4^+^ T cells in the lymph nodes that were Vβ6^+^ (49, 50, 52), although a small but detectable (10% of original percentage) population of Vβ6^+^CD4^+^ T cells remained. However, the percentages of donor-alloreactive Vβ6^+^CD4^+^ T cells both in the lymph nodes and in the thymus (see below) increased again by day +100. Additionally, studies of host-alloreactive donor Vβ3^+^CD4^+^ T cells in one of the MHC-matched models showed a decline in their percentages within CD4^+^ T cells in the lymph nodes at day +10, although there was persistent mixed chimerism in these mice (53).

Regarding the second proposed mechanism, intrathymic clonal deletion, the investigators found in the thymus that donor-alloreactive Vβ6^+^CD4^+^ T cells remained at normal levels at day +14 after cyclophosphamide, at which point there was minimal intrathymic donor chimerism (49, 52). However, Vβ6^+^CD4^+^ T-cell percentages steadily declined thereafter such that they were quite low by day +35 (49, 52), at which point there was low but easily detectable donor chimerism in the thymus. Surprisingly in some mice, donor-alloreactive Vβ6^+^CD4^+^ T cells began to reappear in the thymus at day +70 to +100, which corresponded with loss of substantive intrathymic donor chimerism (49). Interestingly, this loss of donor-alloreactive T-cell intrathymic clonal deletion was not associated with skin allograft rejection (49). Thus, the authors concluded that intrathymic clonal deletion of donor-alloreactive T-cell precursors did occur after cyclophosphamide and required intrathymic mixed chimerism, but was not essential for maintenance of skin allografts at late stages.

The third proposed mechanism, induction of host suppressor T cells, was thought to be the least important of the three and only active at late time points (49). These investigators found that transferring splenocytes at day +14 from tolerized mice to new irradiated mice led to only a short prolongation of skin allograft survival (49). After 100 days, however, transferring splenocytes in this fashion led to marked prolongation of skin allografts (49). This latter effect also was true in models using mice mismatched at both major and minor histocompatibility antigens, even in the absence of any mixed chimerism (54). In their MHC-matched models, antibody treatment to deplete all T cells or just CD4^+^ or CD8^+^ cells suggested that CD8^+^ regulatory T cells were the cells responsible for this effect, since depleting T cells in general or CD8^+^ cells led to more rapid skin allograft rejection (49, 54). However, later work contradicted these findings and instead showed that T-cell or CD4^+^-cell depletion (but not CD8^+^-cell depletion) would obviate the suppressive activity, suggesting instead that CD4^+^ regulatory T cells were responsible (55). These CD4^+^ regulatory T cells mediated suppression in cyclophosphamide-treated mice in an alloantigen-specific manner, which was important since they also showed that donor-alloreactive effector T cells did persist and were not anergic (55).

In their proposed model, there were important interactions between the three mechanisms. As described above, direct peripheral elimination of alloreactive T cells was thought to be the dominant mechanism, followed by intrathymic clonal deletion of alloreactive T-cell precursors. The authors concluded that intrathymic mixed chimerism was necessary for this second step to occur, but intrathymic clonal deletion terminated once mixed chimerism ended. At that turning point, occurring in some mice between days +35 and +100 (49), suppressor T cells became most critical, maintaining a state of immunologic tolerance and preventing skin allograft rejection (49, 54). Playing an adjunct role to suppression, clonal anergy also was suggested to contribute to long-lasting tolerance, since the authors observed no proliferative response in donor-alloreactive T cells that reappeared in the periphery once intrathymic clonal deletion ended (49, 56); however, a similar lack of response also was seen in mice primed with allogeneic MHC-matched splenocytes without cyclophosphamide (49). Furthermore, later findings also showed persistence of donor-alloreactive T cells with the ability to respond to alloantigen and thus not anergic (55), suggesting that the model and its dynamics may be even more complex than initially described.

## Potential Limitations of this Mechanistic Model

Other investigators at the University of Pittsburgh and Johns Hopkins drew on this rich immunologic background to examine the impact of cyclophosphamide given post-transplant in preventing GVHD in HCT models (57–60). This has led directly to the successful clinical translation of this approach in both HCT and in combined solid organ/hematopoietic cell transplantation (6–8, 61, 62).

Yet, the mechanistic model (20) used to explain how cyclophosphamide works clinically largely has been extrapolated from the MHC-matched skin allografting models reviewed above, in which the efficacy of cyclophosphamide was contextual. For cyclophosphamide to prevent skin allograft rejection, the host had to be 6–12 weeks old (46), the transplant had to be MHC-matched (36, 39), a specific dose and type of priming cells had to be used (43), the priming cells had to contain viable stem cells (44), the dose of cyclophosphamide had to be 150–200 mg/kg (43, 44), and a minimal level of mixed chimerism had to be achieved, even if only transiently (45, 52). A single round of cyclophosphamide was unable to overcome the MHC barrier, but instead a two-step approach or inclusion of T-cell-depleting antibodies had to be performed (47, 48).

These models showed that cyclophosphamide led to a decline in the frequency of alloreactive T cells only in the MHC-matched setting. In the MHC-mismatched setting, an initial decline in alloreactive T-cell percentages at day +14 was followed by subsequent rising percentages over the next 3 weeks. The authors conjectured, but did not demonstrate, that highly proliferative T-cell clones would be sensitive to cyclophosphamide, resulting in their elimination, and consequent tolerance induction; conversely, slowly proliferative clones were hypothesized to survive cyclophosphamide treatment in a sensitized state, and thus no tolerance would be induced (63). The authors interpreted the findings of inability of a single dose of cyclophosphamide to induce tolerance to MHC-mismatched skin allografts and selectively eliminate alloreactive T cells as indicating that alloreactivity in the MHC-mismatched setting has slower kinetics than in the MHC-matched setting; in this framework, T cells responding to MHC-mismatched antigens would be less sensitive to cyclophosphamide (39). Such a conclusion is at odds with our broader immunologic understanding that alloreactive T-cell responses are more potent in the MHC-mismatched setting, including clinical manifestations of intense and rapid alloreactivity in the HLA-haploidentical setting that are not observed after HLA-matched HCT (64–66).

Furthermore, the decline in the percentages of donor-alloreactive T cells seen in MHC-matched skin allografting models steadily progressed over several weeks instead of immediately after cyclophosphamide. These kinetics suggest that peripheral deletional tolerance may be operational and may be acting in addition to or instead of direct deletion by cyclophosphamide. Transferring splenocytes at day +14 from tolerized mice to newly irradiated mice led to only minimal prolongation of skin allograft survival, confirming that alloreactive T cells survived cyclophosphamide and retained some functionality (49). Moreover, the reduction in the percentages of alloreactive T cells was shown to be transient and associated with a resurgence of alloreactive T cells that displayed functional impairment (49, 50, 53, 56). Indeed, persistent alloreactive T cells present at 10 weeks after cyclophosphamide could provoke graft rejection in particular circumstances (55). Similarly, splenocytes taken from mice tolerized by PTCy could cause GVHD, inconsistent with the hypothesis of selective alloreactive T-cell elimination. When splenocytes from C3H mice tolerized by cyclophosphamide were given as donor cells for HCT into the same strain used for the priming splenocytes (AKR), no GVHD was induced. But, use of the reverse model (AKR → C3H) resulted in chronic GVHD reactions (67). Likewise, fatal GVHD occurred, albeit at a slightly delayed interval, when cyclophosphamide-treated cells from MHC-mismatched donor/recipient combinations were used (67). Overall, decreases in alloreactive T cells in lymph nodes were observed in some contexts after cyclophosphamide, but were not shown to be due to direct destruction by cyclophosphamide nor were they directly linked mechanistically to prevention of skin allograft rejection.

Although the investigators demonstrated that intrathymic clonal deletion occurred, this also was not strongly linked experimentally with prevention of skin allograft rejection, but rather correlated with mixed chimerism. Thymectomy could worsen outcomes in this model, but half of thymectomized mice still maintained the graft long-term. Moreover, when it occurred in thymectomized mice, rejection generally happened later (42), and thymectomy only had an impact in MHC-matched models (41, 42). Notably, intrathymic clonal deletion could break down at later stages and tolerance to the skin allograft would still be maintained (49), drawing into question the importance of this mechanism for prevention of skin allograft rejection. Also, in some model systems, intrathymic mixed chimerism was not necessary (54). Finally, the role of regulatory T cells was established but there was discrepancy between studies on the relative role of CD4^+^ vs. CD8^+^ regulatory T cells (49, 55).

Ultimately, it is unclear whether some of the elements of the model are mechanistic vs. epiphenomena. Moreover, it is unknown what findings are specific for cyclophosphamide's effects only in the context of this specific model vs. being broadly applicable to other contexts. This is of particular importance given the differential effects seen in these skin allografting models between transplantation of cells vs. solid organs and between MHC-matched vs. MHC-mismatched allografts and the fact that cyclophosphamide has proven efficacy clinically for partially HLA-mismatched HCT (6, 9).

## TOWARD a New Understanding

Despite the limitations noted above, a mechanistic model largely based on extrapolation from the skin allografting models was developed to explain how PTCy prevents GVHD (20, 68); this mechanistic model has since become entrenched in the HCT field. Yet, there are important inconsistencies between what would be predicted from this model and what actually has been observed clinically after HCT. PTCy is most effective at preventing chronic GVHD and the progression of moderate (grade II) to severe (grade III-IV) acute GVHD (6). However, grade II acute GVHD frequently occurs in PTCy-treated patients at rates of 20–80% (6, 15, 16, 69, 70), which would suggest that alloreactive T cells persist after PTCy and are not anergic. Nonetheless, only 10–20% of patients develop chronic GVHD (6, 15, 16, 69, 70), indicating no ongoing clinical alloreactivity even though alloreactive T cells may persist. Furthermore, PTCy is broadly effective in HCT patients across an array of recipient ages, cell doses, HLA-matching, and PTCy doses (6, 9, 69, 71–73), contrasting with the specific requirements needed in the MHC-matched skin allografting studies. Lastly, treatment with a CNI prior to cyclophosphamide blocked cyclophosphamide's efficacy in the skin allografting models (74), but clinically CNIs can be integrated prior to PTCy without any loss, and potentially even improvement, in prevention of GVHD after HCT (75, 76). Therefore, we began to question how well the proposed mechanistic model extrapolated from the MHC-matched skin allografting models (20, 49) serves as an accurate depiction of how PTCy prevents GVHD after allogeneic HCT.

In the skin allografting models, it was posited that regulatory T cells only serve a later role to compensate for breakdown of clonal deletion and reemergence of alloreactive T cells. Yet, given the apparent persistence of alloreactive T cells clinically, we explored the role of CD4^+^ regulatory T cells (Tregs) in HCT in two studies. First, we found that CD4^+^ Tregs recovered rapidly after HCT in patients despite protracted total CD4^+^ T-cell lymphopenia (21). Additionally, we observed that human CD4^+^ Tregs were more resistant to PTCy in mixed lymphocyte cultures *in vitro* and increased expression of aldehyde dehydrogenase (ALDH), the major *in vivo* detoxifying enzyme for cyclophosphamide, both *in vivo* and *in vitro* (21). Beyond just better survival and reconstitution, CD4^+^ Tregs were necessary early post-transplant for GVHD prevention by PTCy in both xenogeneic and MHC-matched HCT models (21, 22), and thymically-derived natural CD4^+^Foxp3^+^ Tregs, rather than peripherally induced CD4^+^Foxp3^+^ Tregs, appeared to be playing the major role in the murine MHC-matched HCT models (22). These findings overall solidified a role for Tregs that seemed more critical than previously ascribed and more important at earlier time points. Notably, given that Tregs were necessary immediately post-HCT for GVHD prevention by PTCy (21, 22), these results implied that alloreactive T-cell elimination either was not occurring or was not as complete after PTCy as had been previously believed.

**Figure 1:**
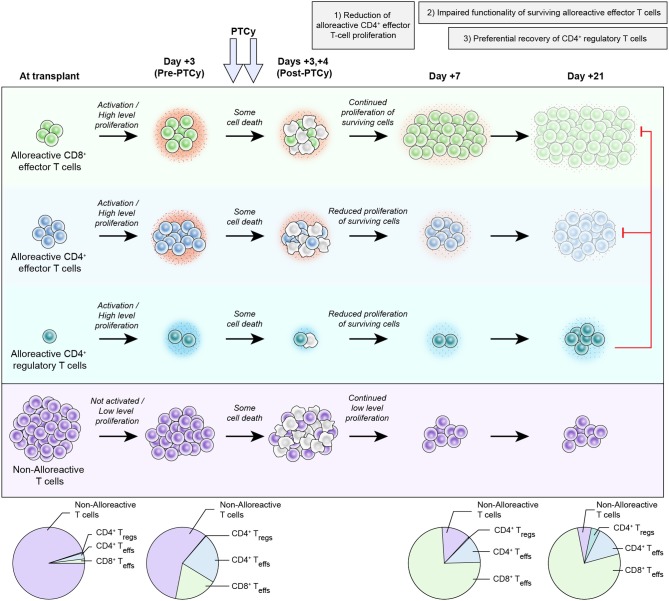
Proposed new working model of the mechanisms by which post-transplantation cyclophosphamide prevents graft-versus-host disease after allogeneic hematopoietic cell transplantation. After infusion into the recipient, host-alloreactive donor T cells become activated, highly proliferative, and more productive of inflammatory cytokines (depicted by the small red dots around the cells). Since PTCy, as an alkylator, is not a cell cycle-dependent cytotoxic agent, both host-alloreactive donor T cells (highly proliferative), and host-non-alloreactive donor T cells (lowly proliferative) are affected and there is some cell death in all subsets. It is unclear *in vivo* whether host-alloreactive donor T cells are more resistant to PTCy or just more rapidly reconstitute due to their ongoing rapid proliferation; survival (or death) after PTCy likely is due to a complex array of factors occurring in each individual cell and may be modulated by several interrelated factors (24). Between post-transplant days +3 and +7, there is continued high-level proliferation of host-alloreactive donor CD8^+^ effector T cells and reduced but continued proliferation of the surviving host-alloreactive donor CD4^+^ T cells (effector (Teff) and regulatory (Treg) cells). The reduced proliferation of host-alloreactive donor CD4^+^ effector T cells seems to be important for PTCy's efficacy as dosing schedules that fail to decrease host-alloreactive donor CD4^+^ effector T-cell proliferation are ineffective or suboptimal at preventing GVHD (79). Impaired functionality of surviving host-alloreactive donor effector T cells occurs early after PTCy (as early as post-transplant day +5) and appears to increase with time, as depicted with progressive decreases in cytokine production and changes in color of those subsets. This change in host-alloreactive donor effector T-cell functionality appears to be in part a direct (or at least rapid) effect of PTCy, but also is augmented by preferential reconstitution of donor CD4^+^ regulatory T cells between days +7 and +21, which suppress the host-alloreactive donor effector T cells, further contributing to their impaired functionality. Throughout this entire process, host-non-alloreactive donor T cells are not activated, maintain low-level proliferation, and consequently contract relative to host-alloreactive donor T cells. The dynamics of host-alloreactive vs. host-non-alloreactive donor T cells at later time points are unknown, but indirect data suggest that they may change with time and/or antigenic (e.g., viral) stimulation (80). This proposed working model is heavily based on experimental data in murine HCT (21–23) and does not account for the integration of other immunosuppressant agents with PTCy as is commonly done clinically. However, the proposed model is expected to be imperfect and incomplete, and further study likely will increase its accuracy, comprehensiveness, and complexity.

Given the primary clinical use of PTCy for HLA-haploidentical HCT, we next developed an MHC-haploidentical HCT model (B6C3F1 → B6D2F1) that parallels clinical HCT to further study the mechanisms underlying GVHD prevention by PTCy (23). We used this model to clarify the three previously proposed mechanisms of GVHD prevention by PTCy: selective elimination of alloreactive T cells, intrathymic clonal deletion of alloreactive T-cell precursors, and induction of Tregs (20, 49). Our primary goal was to test the hypothesis that alloreactive T-cell elimination is a necessary and central mechanism of GVHD prevention by PTCy. In this model, we administered PTCy on days +3 and +4 to further parallel clinical HCT. We first established the optimal PTCy dose (25 mg/kg/day) for GVHD prevention in our model; either lower or higher doses of PTCy were less effective at preventing GVHD and mortality. Alloreactive T cells robustly proliferated post-transplant, consistent with results seen in human HLA-haploidentical HCT (77, 78). CD4^+^ T-cell proliferation was reduced but not eliminated after 25 mg/kg/day PTCy (Figure 1). Surprisingly, CD8^+^ T-cell proliferation was not substantially reduced by 25 mg/kg/day PTCy (Figure 1).

Using Vβ6^+^ as a marker of host-alloreactive donor T cells (akin to what was done in the skin allografting models), we observed that host-alloreactive donor T cells persisted at percentages similar to or even higher than seen in mice not treated with PTCy. The persistence of Vβ6^+^ host-alloreactive donor T cells after PTCy was demonstrated in this model at days +7, +21, and +200 and at specific time points in three other murine HCT models: at day +7 in another MHC-haploidentical model (B6 → B6D2F1), at days +6 and +200 in an MHC-mismatched model (C3H → B6D2F1), and at day +7 in an MHC-matched model (C3H → AKR), and the persistence of Vβ3^+^ and Vβ5^+^ host-alloreactive donor T cells was seen at day +7 in a third MHC-haploidentical model (B6 → B6C3F1) (23).

To additionally confirm that PTCy was not selectively eliminating alloreactive T cells, two other markers of host-alloreactive donor T cells were used: 2C T-cell receptor (TCR)^+^ CD8^+^ T cells and 4C TCR^+^ CD4^+^ T cells. 2C TCR^+^ T cells from B6C3F1 [(C3H x 2C TCR^+^ B6)F1] mice were admixed with wild-type T cells to generate allografts containing a fixed percentage (8%) of the CD8^+^ T cells having the 2C TCR; splenocytes containing this mixture were used as the donor cells for HCT. A similar approach was used for studying 4C TCR^+^ T cells as a percentage (8%) of CD4^+^ T cells. 2C TCR^+^ CD8^+^ host-alloreactive donor T cells remained highly proliferative despite PTCy and actually expanded from 8% of donor CD8^+^ T cells at transplant up to 30–80% of donor CD8^+^ T cells by day +7 (Figure 1) and still were detectable at day +200 (23). Yet, PTCy remained effective at GVHD prevention in this model. 4C TCR^+^ T cells also persisted after PTCy at similar to higher percentages as in vehicle-treated mice, but their proliferation after PTCy was reduced, as we had seen for Vβ6^+^ host-alloreactive donor CD4^+^ T cells (23). In these studies, it was not possible to definitively separate the relative resistance of different T-cell subsets to PTCy vs. changes in early reconstitution after PTCy, particularly given how rapidly host-alloreactive donor T cells were proliferating despite PTCy (and thus numerically expanding) and the *in vivo* system that limited exhaustive tracking of whole-body alloreactive T-cell numbers; regardless, host-alloreactive donor T cells were not selectively eliminated, and if anything, appeared to dominate early reconstitution after PTCy (Figure 1).

To test the fate of host-non-alloreactive donor T cells after HCT, we used our admixed 2C TCR^+^ approach in an MHC-haploidentical model B6 → B6C3F1, wherein 2C TCR^+^ T cells are host-non-alloreactive. In this setting, 2C TCR^+^ T cells maintained a naïve phenotype and low-level proliferation (Figure 1) (23). Thus, since host-alloreactive donor T cells continued to proliferate rapidly and expand despite PTCy, the percentage of CD8^+^ T cells that were host-non-alloreactive 2C TCR^+^ actually greatly contracted (Figure 1). These results demonstrated that GVHD prevention could be achieved by PTCy despite persistence and even expansion of host-alloreactive donor T cells, whereas host-non-alloreactive donor T cells appear not to be the dominant population early after PTCy (Figure 1) as had been previously believed (20). This differs from the previously proposed model wherein antigen-activated donor T cells with high proliferation were proposed to be preferentially targeted by PTCy, whereas non-alloreactive donor T cells were believed to be much less affected, leading to an immune reconstitution devoid of alloreactive T cells (20).

If host-alloreactive donor T cells persist and even expand after PTCy, how is GVHD being mitigated? We examined this question in two different ways (23). The first approach isolated liver-infiltrating donor cells from mice treated with either vehicle or 25 mg/kg/day PTCy and re-stimulated them *in vitro* with alloantigen. We found that PTCy-treated T cells continued to proliferate, but did so to a lesser degree than vehicle-treated mice. Although the effect of PTCy on proliferation was more modest, PTCy-treated cells also produced dramatically less inflammatory cytokines (Figure 1). Similar findings of decreased proliferation and cytokine production were seen when using donor T cells that were flow cytometrically devoid of CD4^+^CD25^+^ T cells to isolate the effect on host-alloreactive donor T cells themselves, rather than the confounding effect of the presence of donor Tregs in the cultures. These results suggested that PTCy was leading to intrinsic functional impairment of host-alloreactive donor T cells (Figure 1). We confirmed these findings by using our 2C TCR^+^ admixed model described above, treating mice with vehicle or 25 mg/kg/day PTCy on days +3 and +4, flow cytometrically sorting viable 2C TCR^+^ CD8^+^ T cells on day +5, and reinfusing them into new irradiated mice which were not further treated. In these serial transplants, mice transplanted with PTCy-treated 2C TCR^+^ T cells had better weights and clinical scores than mice transplanted with vehicle-treated 2C TCR^+^ T cells, despite similar persistence of 2C TCR^+^ T cells numerically at day +150. These results confirmed that PTCy appears to be rapidly inducing functional impairment of surviving alloreactive T cells that contributes to GVHD prevention (Figure 1). Although the nature of this functional impairment has not yet been fully characterized, both the *in vitro* and *in vivo* data above showed that host-alloreactive donor T cells treated with PTCy do continue to respond to alloantigens, albeit at a reduced level, suggesting that they are not becoming anergic.

The second mechanism previously proposed to underlie PTCy's efficacy in preventing skin allograft rejection was intrathymic clonal deletion of alloreactive T-cell precursors. However, in our MHC-haploidentical HCT model (B6C3F1 → B6D2F1), we demonstrated that 25 mg/kg/day PTCy on days +3 and +4 remained effective in thymectomized recipients with no apparent difference in outcomes between mice that were or were not thymectomized (23). These results disproved that the thymus plays a necessary role in GVHD prevention by PTCy. Furthermore, PTCy-treated mice transplanted in these models quickly converted to full donor chimeras (23), demonstrating that mixed chimerism is not necessary to achieve GVHD prevention by PTCy.

The third mechanism previously proposed was the induction of suppressor T cells (49, 54). Our previous data showed that CD4^+^Foxp3^+^ Tregs rapidly reconstituted in HCT patients by a month after PTCy, preferentially survived PTCy *in vitro*, and were necessary for GVHD prevention immediately after PTCy in xenogeneic and MHC-matched HCT models (21, 22). We further investigated the impact of CD4^+^CD25^+^Foxp3^+^ Tregs in our MHC-haploidentical model. We were surprised to find that the percentages of donor CD4^+^ T cells that were CD25^+^Foxp3^+^ were similar or lower at day +7 (after PTCy on days +3/+4) compared with vehicle-treated mice (23); these results were different from what we had observed in human or mouse *in vitro* mixed lymphocyte cultures (21, 22), in which the percentages of Tregs were increased at day +7. Even so, in our MHC-haploidentical HCT model, donor CD4^+^CD25^+^Foxp3^+^ Tregs preferentially reconstituted by day +21 in all four tested organs in mice treated with 25 mg/kg/day PTCy on days +3 and +4 (Figure 1) (23). This effect appeared dose-dependent as mice treated with too low (5 mg/kg/day) or too high (100 mg/kg/day) PTCy doses did not have preferential donor Treg recovery. The lack of preferential recovery of Tregs after the 100 mg/kg/day PTCy dose was not due to an inadequate recovery of Tregs, as the numbers were similar with 25 mg/kg/day PTCy-treated mice, but rather due to a much more robust rebound of host-alloreactive donor effector T cells after 100 mg/kg/day PTCy; these findings may explain the worse GVHD seen histopathologically with that higher dose (23). Thus, the ability of PTCy to constrain host-alloreactive donor effector T-cell proliferation seemed to be optimal after the 25 mg/kg/day dose on days +3 and +4.

Our results provided a few additional novel insights into the role of donor Tregs in GVHD prevention by PTCy. Alloantigen-specific donor Tregs were increased in the liver of PTCy-treated mice at day +21 compared with vehicle-treated mice, and the Treg content of the liver at day +50 correlated very well with the histopathologic score of GVHD in those organs (23). Furthermore, Foxp3^+^ Treg depletion in our MHC-haploidentical model (B6C3F1 → B6D2F1) demonstrated a time dependency. Although they were necessary early post-transplant as we also had shown in our other models (21–23), donor Tregs appeared to play an increasingly important role as time progressed post-transplant; much higher mortality and more rapid and severe GVHD induction were observed when Tregs were depleted at later time points (day +30 and especially day +60 or +150) (23). We also explored whether donor Tregs were sufficient to prevent GVHD in our model as they were in MHC-matched murine HCT or human T-cell-depleted HLA-haploidentical HCT (81, 82). We tested this in our T-cell-replete MHC-haploidentical HCT model by giving CD4^+^CD25^+^ donor Tregs 4 days prior to HCT; GVHD lethality was delayed, but ultimately these mice still developed severe and fatal GVHD, suggesting that donor Tregs, while necessary for GVHD prevention by PTCy, may not be sufficient to prevent GVHD after T-cell-replete MHC-haploidentical HCT (23).

We further tested the role of suppressive mechanisms in GVHD prevention by PTCy in another set of experiments in which mice were transplanted with our B6C3F1 → B6D2F1 MHC-haploidentical HCT model and at a later date had new donor splenocytes infused. Consistent with our findings that PTCy does not work via alloreactive T-cell elimination and that suppressive mechanisms are critical, infusion of new donor splenocytes at various time points (day +5, day +28, day +126, day +150, day +200) did not cause GVHD (23). In fact, mice reinfused on days +5 or +28 were indistinguishable from mice not reinfused with new donor splenocytes. These results suggest that suppressive mechanisms are induced immediately after PTCy and are sufficient to prevent new donor T cells from causing GVHD. We are actively exploring the relevant role of donor Tregs vs. other suppressive cell populations in mediating this effect and whether there may be other immunosuppressive players involved in GVHD prevention by PTCy. Thus, the model proposed in Figure 1 may really serve as a starting place from which to build and refine a complete mechanistic model of GVHD prevention by PTCy. This goal is a major focus of ongoing investigations in our laboratory.

As we increasingly have come to recognize that the previous paradigm of understanding for how cyclophosphamide prolongs MHC-matched skin allograft survival may not apply to GVHD prevention by PTCy, we also have begun to question what we thought we knew about how PTCy should best be applied clinically. As we have described above, the maximal efficacy of PTCy in the MHC-matched skin allografting models was achieved with a high dose (200 mg/kg) given on day +2 or +3 (43). Murine HCT models, building on the skin allografting data, used the 200 mg/kg dose on day +2 or +3 to decrease graft rejection and GVHD (57–60). When PTCy then was translated to patients (8), it was given at 50 mg/kg [close to the maximum tolerable dose in humans and a dose which had showed efficacy in aplastic anemia treatment (83)] and was given on day +3 to space it as far away from conditioning as possible aiming to decrease toxicity. Results from the first phase II study suggested that adding a second dose of PTCy on day +4 might lead to less extensive chronic GVHD than dosing on day +3 only (7); thus, nearly all subsequent studies have used dosing of PTCy at 50 mg/kg/day on days +3 and +4. Our studies in murine MHC-haploidentical and MHC-mismatched HCT models showed that an intermediate dose of PTCy of 25 mg/kg/day on days +3 and +4 appeared superior to both lower and higher doses (23). Consequently, we have extensively studied the impact of the timing and dosing of PTCy on its efficacy in preventing GVHD in our murine MHC-haploidentical HCT model (79). We found that the dose, timing, and cumulative exposure of PTCy all impacted substantially on how well it prevented GVHD and that there were interactions between these three parameters (79). Ultimately, the peak efficacy of PTCy appeared to be at approximately day +4, with dosing at earlier or later time points being less effective; this finding was most pronounced when using suboptimal doses of PTCy (79). Furthermore, the most effective dosing schema of PTCy both reduced host-alloreactive (Vβ6^+^) donor CD4^+^CD25^−^Foxp3^−^ effector T-cell proliferation at day +7 and allowed preferential donor CD4^+^CD25^+^Foxp3^+^ regulatory T-cell reconstitution at day +21, which together may serve as potential biomarkers of effective GVHD prevention by PTCy (79). Compared with PTCy dosing on days +3/+4, dosing on days +1/+2 did not reduce host-alloreactive donor CD4^+^ effector T-cell proliferation at day +7 as effectively, while dosing on days +5/+6 or +7/+8 hindered preferential donor Treg recovery at day +21 (79). Based on these data, we are currently exploring in a clinical trial whether the dosing and timing of PTCy can be optimized in HLA-haploidentical HCT (NCT03983850) with the goals of further improving hematopoietic and immune reconstitution after HCT and reducing toxicity, while maintaining or potentially even improving prevention of acute and chronic GVHD.

## Immunologic Insights from Clinical HCT

As described earlier, we have shown that activated Tregs reconstitute rapidly in patients post-transplant, recovering close to donor levels within a month after HCT despite prolonged CD4^+^ T-cell lymphopenia (21). Another group showed that higher percentages of Tregs at day +14 were associated with decreased risk for acute GVHD (84), while we showed that the percentages of Tregs at days +30 and +60 actually were elevated in patients with active acute GVHD (21), suggesting a potential compensatory increase in those patients. However, these studies have focused on Tregs as a bulk population, and alloantigen-specific Tregs have not been studied in HCT patients treated with PTCy.

The identification of alloreactive effector or regulatory T cells in humans is complicated. Unlike in mice, where we can know that a specific T-cell clone is alloreactive in a specific setting, in humans we generally have to rely on functional characteristics associated with a cell to prove it as an alloreactive clone. Alloreactive T cells are generally expected to derive largely from the naïve T-cell pool, wherein each clone would be expected to exist as a single cell or only a few cells. Thus, it can be difficult to track the fate of alloreactive T cells post-transplant due simply to the inadequacy of sampling; indeed, clones that are <0.01% of T cells cannot be reliably detected on repeat sampling even from the same sample that is split into two halves for T-cell receptor sequencing (80). Furthermore, apparent alloreactive T-cell clones found within GVHD target tissues are not always found in the blood of the same patients (80, 85).

Even so, we recently studied immune reconstitution by flow cytometry and TCR sequencing in patients treated with HLA-matched HCT using PTCy as single-agent GVHD prophylaxis (80). Despite complete or near-complete donor chimerism (15), surprisingly, the TCR repertoires in patients at 1 month post-transplant bore little resemblance to their donors' TCR repertoires (80). In fact, T-cell clones that were expanded in donors were generally undetectable in recipients at 1–2 months post-transplant, whereas frequent donor T-cell clones in patients at 1–2 months post-transplant were generally not able to be tracked back to their origin within the donor repertoires (80); importantly, these patients were older and had been heavily pre-treated, with minimal recent thymic emigrants at these time points. This implied that the repertoire early post-transplant indeed may be largely derived from rare, presumably naïve, clones found in the donor. Indeed, other groups have reported that T-cell reconstitution early after PTCy appears to be coming predominantly from naïve-derived T cells that assume a stem-cell-memory-like phenotype (77, 78). Conversely, memory T-cell clones in the donor, particularly those reactive to pathogens like cytomegalovirus, were not found at high levels early post-transplant, but began to dominate the T-cell repertoire after 3 months post-transplant, at which time point the TCR repertoire in the recipient became increasingly similar to the donor (80). Overall, these results in patients are completely consistent with the new proposed working model (Figure 1), in which expansion of alloreactive T cells, derived in patients from rare donor T cells, occurs early post-transplant despite PTCy. However, the human studies have not yet linked these naïve-derived T-cell clones present early post-transplant to be specifically alloreactive.

Although T cells have been the primary focus of these studies and the proposed mechanistic model, the impact of PTCy on other immune cells has been investigated. Both B cell and natural killer (NK) cells appear largely to turn over post-transplant, as the cells that do recover tend to be predominantly naïve mature B cells and immature NK cells (80, 86). However, there are no data available regarding any potential role of either of these subsets in acute or chronic GVHD prevention by PTCy. In patients treated with PTCy and bortezomib for GVHD prophylaxis, dendritic cells isolated early post-transplant had decreased expression of co-stimulatory and maturation markers (87). T cells stimulated with these dendritic cells were less proliferative than T cells stimulated with dendritic cells derived from patients treated with standard CNI-based GVHD prophylaxis (87). However, it is unclear what of this effect is due to PTCy vs. bortezomib and whether this is mechanistic or an epiphenomenon.

## Immunologic Insights From Clinical Solid Organ Transplantation

The success of clinical solid organ transplantation is limited by absence of tolerance induction in the vast majority of patients, generally requiring long-term immunosuppression to prevent allograft loss. As an alternative, investigators have pursued the addition of HCT to solid organ transplantation with the goal of inducing long-term tolerance and thus the ability to reduce or remove immunosuppression. Given PTCy's efficacy clinically in HCT and preclinically in skin and other solid organ transplantation models (19, 57, 58, 88), PTCy has been incorporated into some approaches to HLA-mismatched or HLA-haploidentical combined HCT/kidney transplantation (61, 62, 89, 90); the results have been promising with very low rates of GVHD and the ability to fully remove immunosuppression in all patients with persisting donor chimerism except for one patient with chronic GVHD (89, 90). However, in patients without durable donor chimerism, graft rejection could occur even when hyporesponsiveness of recipient cells to donor cells *in vitro* was observed (62). These data suggest that mixed chimerism was protective, but that the T cells present after PTCy still could mediate graft rejection.

Morris and colleagues have proposed an *in vitro* method to screen for donor-alloreactive T cells, wherein mixed lymphocyte reactions were performed between donor and recipient PBMCs, followed by flow cytometric sorting of the T cells reactive to the donor antigens (91). Deep sequencing of the TCRβ repertoires was performed in order to identify the presumptive alloreactive T cells (those proliferating in response to alloantigen) and evaluate the fate of these clones after transplant. Six patients were studied. Even in PTCy-treated patients who were functionally tolerant, anti-donor T-cell responses could be seen persisting for 6–18 months post-transplant. Although a decrease in donor-reactive T-cell clones was observed post-transplant, overall the reduction was modest and progressive over 6–18 months post-transplant (91). This did differ from the two patients studied who were not treated with PTCy, in which progressive increases in the number of donor-reactive CD4^+^ T-cell clones were seen. Overall, these results support that alloreactive T-cell deletion may be occurring to a limited extent in patients after combined HCT/kidney transplantation using PTCy. Yet, this does not appear to be an immediate effect of PTCy but rather a progressive change over months to years, reflecting likely peripheral deletional tolerance in these mixed chimeric states.

## Discussion

The use of cyclophosphamide for inducing tolerance to skin allografting models had been thought to rest on three principles, with the primary mechanism being selective elimination of alloreactive T cells by PTCy (49, 53). Although a preferential reduction of alloreactive T cells over time was shown in MHC-matched skin allografting models, it was not shown in MHC-mismatched models. Furthermore, the slow reduction of alloreactive T-cell percentages after cyclophosphamide was not directly linked to killing by cyclophosphamide. Minimal levels of mixed chimerism were an essential component that consistently tracked with alloreactive T-cell depletion in those studies. Indeed, we have observed peripheral deletion of alloreactive T cells in thymectomized mice treated with T-cell-depleted HCT without PTCy (23). Thus, it is unclear whether the reduction of alloreactive T cells seen in the MHC-matched skin allografting models was directly or indirectly related to cyclophosphamide. Even if that effect was directly related to cyclophosphamide, the effectiveness of cyclophosphamide in those models was contextual, including differential effects on tumor vs. skin allografts and on MHC-matched vs. MHC-mismatched allografts, raising concerns about the relevance of such a model for MHC-haploidentical HCT (Table 1). Indeed, clinically PTCy is highly effective across an array of donor types, transplant platforms, graft compositions and cell doses, and recipient and donor ages (6). In our recent paper (23), we tried to replicate some of the contextuality of the skin allografting models by exploring different doses of PTCy, the inclusion or exclusion of radiation prior to HCT, and investigation of MHC-matched, MHC-haploidentical, and fully MHC-mismatched models, but in all cases we saw persistence of host-alloreactive donor T cells in the recipients at percentages that were similar to or even higher than were transplanted (23).

**Table 1:** Elements of the previously proposed mechanistic model as relates to outcomes observed with experimental murine skin allografting or allogeneic HCT.

	**Skin grafting**	**Hematopoietic cell transplantation**
Alloreactive T cells	• The percentages of donor-alloreactive CD4^+^ T cells were selectively reduced between days 0 and +35 in MHC-matched models, increasing again afterwards. This was associated with abrogation of alloreactive functional responses. • Host-alloreactive donor CD4^+^ T cells were selectively reduced at day +10 in MHC-matched models. • There was not a sustained decrease in donor-alloreactive CD4^+^ T cells in MHC-mismatched models. This was associated with persistence and early increases in alloreactive functional responses. • The reduction of donor-alloreactive CD4^+^ T cells in MHC-matched models was not linked mechanistically with the fate of skin allografts. • Donor-alloreactive CD4^+^ T cells present at later time points were in many cases anergic *in vitro*; when not anergic, they still had reduced functionality. Even so, these alloreactive T cells retained sufficient functionality to cause graft rejection or GVHD in serial transplants and also could cause graft rejection when regulatory T cells were removed.	• No selective elimination of host-alloreactive donor T cells was observed at early or late time points. • The lack of host-alloreactive donor T-cell elimination was seen in MHC-matched, MHC-haploidentical, and MHC-mismatched HCT models. • Persistence of host-alloreactive donor T cells was seen regardless of the dose of PTCy used or whether the mice were irradiated. • GVHD prevention occurred despite host-alloreactive donor T-cell persistence. • PTCy at the optimal dose had minimal impact on host-alloreactive donor CD8^+^ T-cell proliferation but did reduce host-alloreactive donor CD4^+^ T-cell proliferation. • 2C TCR^+^ donor T cells preferentially expanded despite PTCy in a model wherein they were host-alloreactive, but contracted in a model wherein they were host-non-alloreactive. Thus, host-alloreactive donor T cells appeared to preferentially reconstitute post-transplant due to their survival and continued proliferation after PTCy. • Host-alloreactive donor T cells did have reduced functionality after PTCy in terms of their *in vitro* proliferation and cytokine production in response to alloantigen and their *in vivo* ability to cause GVHD in that mouse or on serial transfer. Yet, these alloreactive T cells were not functionally or phenotypically anergic. • Host-alloreactive donor T-cell elimination was seen in thymectomized mice treated with T-cell-depleted HCT without PTCy, suggesting that peripheral deletion of alloreactive T cells can occur independently of PTCy.
Intrathymic clonal deletion of alloreactive T-cell precursors	• Intrathymic clonal deletion occurred only in settings wherein cyclophosphamide was effective, but was not linked mechanistically with skin graft rejection. • Intrathymic clonal deletion was linked in most cases with intrathymic mixed chimerism that was at least transient in nature. • Thymectomy decreased survival of skin allografts in a subset of mice in MHC-matched models, but had no impact in MHC-mismatched models. • The breakdown of intrathymic clonal deletion was associated with loss of intrathymic mixed chimerism, but skin allografts were not rejected when this occurred.	• The thymus was not necessary for GVHD prevention by PTCy. • Outcomes were similar between thymectomized and non-thymectomized mice treated with PTCy. • Full donor chimerism was rapidly achieved in PTCy-treated mice. Of note, mice treated with TCD BM only had persistent mixed chimerism within T cells despite the myeloablative conditioning intensity.
Suppressor cells	• Depletion of suppressor T cells at late time points reduced allograft survival. There were mixed data regarding whether depletion of CD4^+^ or CD8^+^ T cells mediated this effect. • The transfer of splenocytes at day +14 from mice tolerized by cyclophosphamide only slightly prolonged skin allograft survival, whereas it led to a much greater prolongation of survival when performed at day +100. • The suppression exerted by CD4^+^ Tregs was mediated in an alloantigen-specific manner.	• CD4^+^CD25^+^Foxp3^+^ donor T cells, including those that were alloantigen-specific, preferentially reconstituted after PTCy. • CD4^+^CD25^+^Foxp3^+^ donor T cells played a necessary role in GVHD prevention by PTCy, but did not appear sufficient to prevent severe and fatal GVHD. • Foxp3^+^ donor T cells were necessary for GVHD prevention by PTCy both at early and late post-transplant time points, but appeared increasingly important as time progressed. • The suppressive mechanisms induced early after PTCy were sufficient to prevent new donor T cells from causing GVHD.

Our current understanding of how PTCy works to prevent GVHD has greatly evolved over the past several years. The initial proposed mechanistic model extrapolated from the skin allografting models had posited that PTCy works primarily through selectively eliminating alloreactive T cells (68). Subsequently, our work in showing a necessary role for Tregs confirmed the role of Tregs identified in the skin allografting models; therefore, the model was revised to include the preferential recovery of Tregs and an important balance between effector and regulatory T cells (20). Additionally, intrathymic clonal deletion was added back into the model to reflect the initial MHC-matched skin allografting data (20, 49). Our recent work has affirmed the role of Tregs, but showed that neither alloreactive T-cell elimination nor intrathymic clonal elimination are necessary for GVHD prevention by PTCy (23). Rather, PTCy appears to induce functional impairment of alloreactive T cells, which is supported by the rapid induction of active suppressive mechanisms after PTCy. These suppressive mechanisms include the preferential recovery of Tregs, including those that are alloantigen-specific. Ongoing work in the laboratory suggests that the model may be even more complicated than that shown in Figure 1, but the presented working model at least displays our level of understanding at this time.

This current understanding fits much better with clinical observations after PTCy than the prior model. PTCy is effective in older and heavily pretreated patients, many of whom lack substantive thymic function (6). Clinically, acute GVHD occurs frequently after PTCy and actually is associated with improved outcomes in those patients (69, 92), consistent with persistence of alloreactive T cells after PTCy. Yet, severe acute or chronic GVHD is infrequent after PTCy, consistent with our model that persisting alloreactive T cells are becoming functionally impaired. GVHD incidence is associated with lower levels of Tregs (23, 84), but clinically Tregs also have been found to be increased in patients with active acute GVHD (21); therefore, Tregs may play roles both in preventing acute GVHD but also controlling breakthrough GVHD and preventing its progression to more severe forms. However, how the integration of adjunct immunosuppression with PTCy, both in terms of the agents used and their timing in relation to PTCy, affects GVHD prevention by PTCy and PTCy's impact on specific immune subsets require further study (93). Furthermore, our recently published work suggests that intermediate, rather than high, dose PTCy may be most effective at preventing GVHD in two murine HCT models (23). Additionally, we have recently demonstrated in our murine MHC-haploidentical HCT model that the optimal timing of PTCy appears to differ from that demonstrated in murine skin allografting models (79). The clinical relevance of these findings concerning the optimal dosing and timing of PTCy requires further study in human HCT, which is being done in a clinical study at our institution.

Improving upon outcomes of patients treated with PTCy may be achieved most rapidly by using a paradigm of understanding that is both based on HCT data and consistent with clinical observations in humans. The persistence of alloreactive T cells after PTCy may allow for the reinduction of a graft-versus-host anti-tumor response in patients with relapsed or aggressive disease post-transplant. Conversely, better understanding the highly active suppressive mechanisms induced by PTCy may allow for better prevention and treatment of GVHD and other post-transplant inflammatory conditions. These mechanisms will provide insight into the pathophysiology of GVHD and its prevention, but also may be directly relevant for improving outcomes in other clinical settings in which tolerance induction is desired, such as autoimmunity and solid organ transplantation. Ultimately, we hope that our recent data and proposed working model will help facilitate the rational development of novel approaches to further reduce GVHD incidence and severity, improve immune reconstitution, and decrease malignancy relapse post-transplant.

## Author Contributions

NN and CK wrote the manuscript and designed the figure.

### Conflict of Interest

The authors declare that the research was conducted in the absence of any commercial or financial relationships that could be construed as a potential conflict of interest.
